# Laboratory Diagnosis of Four Recent Sporadic Cases of Community-acquired SARS, Guangdong Province, China

**DOI:** 10.3201/eid1010.040445

**Published:** 2004-10

**Authors:** Guodong Liang, Qiuxia Chen, Jianguo Xu, Yufei Liu, Wilina Lim, J.S.M. Peiris, Larry J. Anderson, Li Ruan, Hui Li, Biao Kan, Biao Di, Peter Cheng, K.H. Chan, Dean D. Erdman, Shuyan Gu, Xinge Yan, Weili Liang, Duanhua Zhou, Lia Haynes, Shumin Duan, Xin Zhang, Han Zheng, Yang Gao, Suxiang Tong, Dexin Li, Ling Fang, Pengzhe Qin, Wenbo Xu, SARS Diagnosis Working Group

**Affiliations:** * Chinese Center for Disease Control and Prevention, Beijing, People's Republic of China;; † Center for Disease Control and Prevention of Guangdong Province, Guangzhou, People's Republic of China;; ‡ Center for Disease Control and Prevention of Guangzhou, Guangzhou, People's Republic of China;; § Hong Kong Department of Health, Hong Kong Special Administrative Region, People's Republic of China;; ¶ Queen Mary Hospital, Hong Kong Special Administrative Region, People's Republic of China;; # Centers For Disease Control and Prevention, Atlanta, Georgia, USA

**Keywords:** research, SARS, Coronovirus, China

## Abstract

Four cases of severe acute respiratory syndrome (SARS) that occurred from December 16, 2003, to January 8, 2004, in the city of Guangzhou, Guangdong Province, China, were investigated. Clinical specimens collected from these patients were tested by provincial and national laboratories in China as well as members of the World Health Organization SARS Reference and Verification Laboratory Network in a collaborative effort to identify and confirm SARS-associated coronavirus (SARS-CoV) infection. Although SARS-CoV was not isolated from any patient, specimens from three patients were positive for viral RNA by reverse transcription–polymerase chain reaction assay, and all patients had detectable rises in SARS-CoV–specific antibodies. This study shows the effectiveness of a collaborative, multilaboratory response to diagnose SARS.

## Abstract 2

Four sporadic cases of SARS-associated coronavirus infection were identified through collaboration of four laboratories.

In November 2002, cases of a highly contagious and severe atypical pneumonia were identified in Guangdong Province in southern China (*1*). By March 2003, the infection had spread to Hong Kong, Singapore, Vietnam, Taiwan, Canada, and the United States. Designated severe acute respiratory syndrome (SARS), the outbreak was subsequently linked to infection with a previously unrecognized coronavirus (SARS-CoV) (*2**–**4*). The outbreak ended with the World Health Organization (WHO) announcement on July 5, 2003, that the last patient had recovered and the human chain of SARS transmission was broken (*5*). In all, 29 countries reported to WHO >8,000 SARS cases with 774 deaths (*6*).

Since WHO's declaration of the end of the SARS epidemic, 17 cases of laboratory-confirmed SARS-CoV infection have been reported. Infections in 13 patients were associated with laboratories: 6 exposed while working in the laboratory and 7 contacts of a patient with a laboratory-acquired case (*7**–**9*). The other four cases were acquired in the community. On December 26, the Chinese Ministry of Health informed WHO of the first case of community-acquired SARS in Guangzhou, the capital city of Guangdong Province. This case was rapidly followed by reports of three additional cases, all linked to a region in Guangzhou (*10*). Although none of these cases was fatal or resulted in documented secondary transmission, they demonstrate that community-acquired infection with SARS-CoV, and potential reemergence of SARS leading to epidemic spread, remains a possibility. These cases also highlight some issues associated with diagnosing and confirming the diagnosis of SARS-CoV infection. In this report, we describe the laboratory diagnosis and associated confirmation of SARS-CoV infection for these four cases.

## Materials and Methods

### Patients and Specimens

From December 16, 2003, to January 8, 2004, four SARS cases were identified in Guangzhou, Guangdong Province, China. Patient 1 was a 32-year-old male television producer in whom fever and headache developed on December 16, 2003. Patient 2 was a 20-year-old female waitress who became ill on December 26. Patient 3 was a 35-year-old male businessman in whom fever developed on December 30. Patient 4 was a 40-year-old male hospital director and practicing physician who became ill on January 8, 2004. All patients had temperatures >38°C and x-ray evidence of pneumonia. A confirmed case of SARS is defined as clinically compatible illness with laboratory-confirmed evidence of SARS-CoV infection. Initial tests by the Center for Disease Control and Prevention of Guangdong Province and the Center for Disease Control and Prevention of Guangzhou (laboratory A; laboratories that participated in this study are listed in Table 1) were positive for SARS-CoV, which prompted a systematic collection of clinical specimens, including respiratory secretions, urine, stool, and serum collected at different time points in patients' illnesses. Confirmatory testing was performed by the Institute for Viral Disease Control and Prevention and Institute for Communicable Infection Disease, China Center for Disease Control (laboratory B) and members of the WHO SARS Reference and Verification Laboratory Network (*11*), including the Government Virus Unit and Department of Microbiology, Queen Mary Hospital, Hong Kong Special Administrative Region (laboratory C), and the U.S. Centers for Disease Control and Prevention (laboratory D).

**Table 1:** WHO network laboratories that tested specimens from the four SARS patients in Guangdong Province, China^a^

Laboratory code^b^	SARS-CoV						OC43/229E			
	Real-time RT-PCR	RT-PCR Sequencing	EIA	IFA	Neutralization	Isolation	RT-PCR	EIA	IFA	Neutralization
A	√	√	√	√		√				
B	√	√	√		√	√				
C	√	√	√	√	√	√			√	
D	√	√	√		√	√	√	√		√

^a^WHO, World Health Organization; SARS, severe acute respiratory syndrome; CoV, coronavirus; RT-PCR, reverse transcription–polymerase chain reaction; EIA, enzyme immuno assay; IFA, immunofluorescence assay. ^b^Laboratory A, Center for Disease Control and Prevention of Guangdong Province and Center for Disease Control and Prevention of Guangzhou; laboratory B, Institute for Viral Disease Control and Prevention and Institute for Communicable Infectious Disease, Chinese Center for Disease Control and Prevention; laboratory C, Government Virus Unit and Department of Microbiology, Queen Mary Hospital; laboratory D, U.S. Centers for Disease Control and Prevention.

### Serologic Tests

#### SARS-CoV Microneutralization Assay

Serum specimens were tested by laboratories B, C, and D for neutralizing antibodies to SARS-CoV by microneutralization assay using the procedure of Sui et al. (*12*) but with different virus strains (laboratory D, Urbani strain; laboratory B, P9 and P11; laboratory C, strain 6109 or HKU-39846). Briefly, serial dilutions were prepared and added in triplicate to 96-well plates (Costar, Corning, NY). Approximately 75 PFU of SARS-CoV was added to the diluted serum samples and incubated at 37°C for 45 min. Vero E6 cells (2 x 10^5^/mL) were added to the wells, and the mixture was incubated at 37°C for 3 to 4 days. Results were visualized by staining the wells with a crystal violet–formaldehyde staining reagent (0.013% crystal violet, 2.5% ethanol, and 10% formaldehyde in 0.01 mol/L phosphate-buffered saline [PBS]) for 1 h at room temperature. The neutralization titer was measured as the reciprocal of the highest serum dilution that completely inhibited Vero E6 cell lysis in at least two of the three triplicate wells.

#### SARS-CoV Enzyme Immunoassay

Laboratories A, B, and C performed serologic testing for SARS-CoV–specific antibodies by using an indirect enzyme immunoassay (EIA) kit (Beijing BGI-GBI Biotech Co., Beijing, China) in accordance with the manufacturer's instructions. Briefly, diluted serum specimens were added to SARS-CoV lysate–coated wells and incubated at 37°C for 30 min. The wells were rinsed with a wash solution and incubated with a conjugated antibody solution at 37°C for 30 min. After the washing, a substrate solution was added to wells and incubated at 37°C for 10 min in the dark. Termination solution was added and the optical density (OD) measured using 450 nm with a reference wavelength at 630 nm. For every assay, one blank control, one positive control, and two negative control wells were included. The cutoff value for a positive test was defined as 0.13 plus the mean OD of negative control wells. Specimens were considered positive for SARS-CoV antibodies if the calculated value (observed OD readings minus OD reading of the blank control) exceeded the cutoff value.

Laboratory D performed serologic testing for SARS with an in-house indirect EIA (*2*). Briefly, serially diluted serum specimens were added to 96-well microtiter plates (Dynatech Laboratories, Inc., Chantilly, VA) precoated with either γ-irradiated SARS-CoV lysate (Urbani strain) or Vero E6 cell lysate and incubated at 37°C for 1 h. After being rinsed with PBS-Tween-20, plates were incubated with goat anti-human immunoglobulin (Ig) G, IgA, and IgM conjugated to horseradish peroxidase (Kirkegaard and Perry Laboratories, Inc., Gaithersburg, MD) at 37°C for 1 h. After washing, 2,2-azo-bis(3-ethylbenzthiazolin sulfonic acid) (ABTS) substrate (Kirkegaard and Perry Laboratories, Inc.) was added for 30 min at 37°C. ODs were measured at 410 nm and 490 nm wavelength. Specimens were considered positive for SARS-CoV antibodies if the adjusted sum OD (sum of differences between SARS-CoV antigen and control antigen wells) for the 1:100 through 1:6,400 dilutions exceeded 1.25 and the titer of the specimen was >1:400. The titer for a specimen was taken as the highest dilution that had a positive adjusted OD value >0.21.

#### SARS-CoV Immunofluorescence Assay

Laboratory A performed an in-house immunofluorescence assay (IFA). Briefly, SARS-CoV (strain F69)–infected Vero E6 cells spotted and acetone-fixed on glass slides were prepared in advance of testing. Serum samples with a starting dilution of 1:25 were deposited onto the slides and incubated for 30 min at 37°C. The slides were then rinsed and blot-dried, and a fluorescein-labeled polyvalent anti-human immunoglobulin (Biosource International, Camarillo, CA) was added and incubated for 30 min at 37°C. The slides were then rinsed twice, mounted with phosphate-buffered glycerol and coverslip, and examined with a UV microscope. The immunofluorescence titer was taken as the highest dilution that showed a positive reaction (apple green fluorescent cytoplasmic granules). Laboratory C performed a similar IFA but modified it to use SARS-CoV (strain 6109)–infected FRhK4 cells.

#### 229E/OC43 Microneutralization Assay

A microneutralization assay for human coronaviruses 229E and OC43 was developed for this study by laboratory D. Rhabdomyosarcoma (RD) cells were grown to 80%–90% confluence in Dulbecco's Modified Eagle's medium (DMEM) (GIBCO-Invitrogen, Carlsbad, CA) containing 10% defined fetal bovine serum (FBS) (HyClone, Logan, UT). 229E (ATCC VR-740) and OC43 (ATCC VR-759) were introduced onto the washed monolayers, and cultures were incubated at 33°C with 6% CO_2_ for 5 days. The infected cultures were submitted to three freeze-and-thaw cycles, clarified by low-speed centrifugation, and stored at –70°C. Virus stocks were titrated by adding 50 µL of 10-fold dilutions (eight replicates per dilution) of 229E and OC43 to 96-well culture plates (Costar, Cambridge, MA), followed by adding 50 µL of 2 x 10^5^ RD cells/mL and incubating at 33°C for 5 days. Infected wells were identified by an immunofluorescence assay as described elsewhere (*13*) by using monoclonal antibodies specific to 229E or OC43 nucleo protein. The 50% tissue-culture infective dose (TCID_50_) was determined by the method of Reed and Muench (*14*). For the microneutralization assay, 50 µL of heat-inactivated human and animal control hyperimmune serum samples were serially twofold diluted in 10% FBS-DMEM in triplicate wells. Each sample was diluted in duplicate 96-well tissue culture plates followed by adding 100 TCID_50_ of 229E or OC43. After 1 h incubation, 50 µL of 2 x 10^5^ RD cells/mL was added, and plates were incubated at 33°C in 6% CO_2_ for 5 days. A back titration was included in each test. Neutralization titers were defined as the reciprocal of the highest serum dilution that completely inhibited fluorescence in at least two of the three triplicate wells.

#### OC43 Immunofluorescence Assay

Laboratory C performed an in-house IFA as described above for SARS-CoV, but it was modified to use OC43-infected BSC-1 cells.

#### OC43/229E Enzyme Immunoassay

Laboratory D performed serologic testing for human coronaviruses by using an in-house indirect EIA. Briefly, 96-well microtiter plates (Dynatech Laboratories) were coated overnight at 4°C with previously optimized concentrations of clarified lysates of OC43 and 229E and uninfected RD cells as prepared for the microneutralization assay above. Serially diluted serum specimens (1:100 through 1:3,200) were added and incubated for 1.5 h at 37°C. After being washed with PBS-Tween-20, the plates were incubated at 37°C for 1 h with goat anti-human IgG conjugated with horseradish peroxidase (Sigma, St. Louis, MO). After similar washing, a tetra-methyl benzidine substrate (Zymed Laboratories, San Francisco, CA) was added and incubated for 15 min at room temperature. The colorometric reaction was stopped with 2 mol/L phosphoric acid, and ODs were measured in dual wavelength mode at 450 nm and 650 nm. The adjusted sum OD of duplicate wells of the positive and negative antigen was determined for each serum dilution, and the highest dilution showing a >0.1 OD was taken as the virus-specific IgG antibody titer. A titer rise of more than fourfold between early and late serum pairs was considered evidence of recent infection with OC43 or 229E.

### Molecular Studies

#### SARS-CoV Real-time RT-PCR

Respiratory specimens and stool were tested for SARS-CoV RNA by using real-time reverse transcription–polymerase chain reaction (RT-PCR) assays from several different sources. Laboratories A, B, and C used assays developed by Piji Bioengineering (Shenzhen, China) and Artus GmbH (Hamburg, Germany) following the manufacturers' instructions. Laboratory D used an in-house real-time RT-PCR procedure (*15*). Appropriate RT-PCR controls, including positive SARS RNA and negative extraction (water blank), and amplification controls were included in each run. In addition, laboratory D tested each specimen for human RNAse P to ensure the adequacy of RNA extraction and to monitor for RT-PCR inhibitors.

#### OC43/229E RT-PCR

RT-PCR for human OC43 and human 229E was performed on throat swabs by the degenerated consensus PCR primers for the genus *Coronavirus* by using the SuperScript One Step RT-PCR kit (Invitrogen). These primers were derived from a highly conserved region in open reading frame (ORF) 1b of the *pol* gene. Viral total nucleic acid was processed from throat swabs, and RT-PCR was carried out by using the method previously described (*15*).

#### SARS-CoV Sequencing

Sequences from the 3´ third of the SARS-CoV genome were obtained from overlapping RT-PCR products that covered the envelope (E), membrane (M), and nucleocapsid (N) structural protein genes, plus several other gaps of unknown function, such as S-E gap between S ORF and E ORF and M-N gap between S ORF and E ORF, by using a previously described method (*16*).

### Culture

Virus isolation was attempted on RT-PCR–positive respiratory specimens collected from patients 1 and 2 by methods previously described (*2*). Briefly, 100 µL of antimicrobial drug–treated specimen was introduced into tube cultures of Vero E6 cells and incubated at room temperature for 1 h. Fresh modified DMEM with 2% fetal calf serum was added, and cultures were incubated at 37°C with rocking. Cultures were observed daily for cytopathic effect for 2 weeks then blind passaged. Negative cultures for SARS-CoV were confirmed by RT-PCR as described.

## Results

### Serologic Testing

All but one of the serum specimens from these patients tested positive for SARS-CoV antibodies by all laboratories using multiple assay formats, including EIA, IFA, and neutralization assay (Table 2). All four patients had detectable SARS-CoV antibodies by one or more laboratories very early in the illness; serum specimens collected 6 days after onset from patients 1 and 2 were positive by all laboratories by one or more methods, and specimens collected at 8 days from patients 3 and 4 were positive by EIA performed at laboratories A and B, respectively. Where comparisons could be made, the pattern of antibody responses were similar for all assays, and a fourfold or greater rise in EIA or IFA antibodies was demonstrable in multiple laboratories in three of the four patients. A fourfold rise in SARS-CoV antibodies in patient 3 was identified by only one laboratory (laboratory A) by IFA; laboratory A was the only laboratory that tested the earliest specimen from patient 3 and tested the serum specimens as they arrived and not concurrently.

**Table 2:** SARS-CoV EIA, IFA, and neutralization test results for the four SARS patients in Guangdong Province, China^a^

Patient	No. days^b^	EIA				IFA		Neutralization		
		A	B	C^c^	D	A	C	B	C^d^	D
1 (onset 12/16/2003)	6	4	160	+	400	10	25	16	10	20
	7	16	320	+	1,600	80	200			
	8	32	640			160				
	9	128	1,280							
	10	128	1,280			160				
	11	128	1,280	+	6,400	160	200			
	12	128	1,280					64	160	
	13	128	1,280							
	15	64		+	6,400		400			
	22	64			6,400					160
2 (onset 12/26/2003)	6		10			10		<8	40	
	7	4	20	+		80	100			
	8	8	40		1,600	160				160
	9		80			160				
	10		80			160				
	11	16			6,400	160				
	17			+	6,400		1,600	16	160	
	19	16								
	21				6,400					160
	22	16								
3 (onset 12/30/2003)	8	16								
	9		160		6,400	20				160
	10	16	320	+		80	200		80	
	11	64	320			320				
	13	16		+	6,400		400	32	160	
	17	32			6,400					160
4 (onset 1/8/2004)	8	–	10				<25		<10	
	11		10							
	15	80	160		1,600	320	1,600			80
	17	160								
	18				1,600				20	
	21	160			1,600					80

^a^SARS-CoV, severe acute respiratory syndrome–associated coronavirus; EIA, enzyme immuno assay; IFA, immunofluorescence assay; +, positive, –, negative. Where no value appears, the test was not performed. ^b^Number of days the specimen was collected after symptom onset. ^c^Specimens tested by Government Virus Unit, Hong Kong Special Administrative Region and Department of Microbiology, Queen Mary Hospital. ^d^Specimens tested by Government Virus Unit, Hong Kong Special Administrative Region, with a subset tested by Department of Microbiology, Queen Mary Hospital.

A concurrent rise in OC43 antibodies was detected by IFA (laboratory C) in patient 4. To assess the possibility of OC43-induced SARS antibodies reacting with SARS-CoV and confounding the diagnosis of SARS, early and late serum specimens from all patients were simultaneously tested by laboratory D for SARS-CoV, OC43, and 229E antibodies by neutralization assay and EIA (Table 3). In these tests, no rises in either EIA or neutralizing antibody titers were noted to OC43 or 229E. The serum pair from patient 1 had a rise in SARS neutralizing antibodies, and the serum pair from patient 2 had a rise in SARS EIA antibodies (Table 2 and Table 3). The earliest acute-phase serum specimens for patients 2–4 were unavailable for these tests. Neutralizing antibody titers were not detected to 229E and were detected at a lower titer to OC43 than to SARS-CoV; previous studies have shown a lack of SARS-CoV antibodies in paired serum specimens from patients with acute 229E and OC43 infections (*2*).

**Table 3:** SARS-CoV, OC43, and 229E neutralization test results for the four SARS cases in Guangdong Province, China^a^

Patient	No. days^b^	SARS-CoV			OC43^c^	229E^c^
		B	C	D	D	D
1 (onset 12/16/2003)	6	16	10	20	20	<20
	12	64	160			
	22			160	20	<20
2 (onset 12/26/2003)	6	<8	40			
	8			160	20	<20
	17	16	160			
	21			160	20	<20
3 (onset 12/30/2003)	9			160	40	<20
	10		80			
	13	32	160			
	17			160	40	<20
4 (onset 1/8/2004)	8		<10			
	15			80	20	<20
	18		20			
	21			80	20	<20

^a^SARS-CoV, severe acute respiratory syndrome–associated coronavirus. When no values appear, the test was not performed. ^b^Number of days the specimen was collected after symptom onset. ^c^OC43 and 229E neutralization tests were conducted by laboratory D only.

### Virus Detection

A variety of specimens were tested for SARS-CoV by culture isolation and for SARS-CoV RNA by multiple real-time RT-PCR assays (Table 4). Although virus was not successfully isolated from any of the respiratory specimens, viral RNA was detected by RT-PCR in several respiratory specimens from patients 1 and 2 by two or more laboratories and by one laboratory from a single stool specimen from patient 4. In contrast, all respiratory specimens were negative for other coronaviruses by RT-PCR. The RT-PCR–positive throat swabs were collected on days 6, 8, and 10 for patient 1 and on days 6 and 8 for patient 2. The amount of viral RNA in these specimens was small, as shown by threshold cycle values >35 with the real-time RT-PCR assays and inconsistent positivity between laboratories for all but the day 6 throat swab from patient 1. None of the other types of specimens, including multiple stool or stool swab specimens on three of the patients, tested positive for SARS-CoV RNA. Only the throat swab collected on day 6 from patient 1 had sufficient viral RNA for the initial sequencing studies. Sequences were confirmed to be SARS-CoV and most closely matched isolates from civet cats taken in November and December from wild animal markets in Guangdong Province (*17*). A more extensive description of these sequences will be presented in a follow-up report.

**Table 4:** SARS-CoV real-time RT-PCR test results for the four SARS patients in Guangdong Province, China^a^

Patient	No. days^b^	Specimen type	Real-time RT-PCR			
			A	B	C^c^	D
1 (onset 12/16/2003)	6	Throat swab	+	+	+	+
	7	Stool	–	–	–	–
		Sputum	–	–	–	–
	8	Throat swab	+	–	–	–
		Stool	–	–	–	–
		Serum	–	–	–	–
	9	Throat swab	–	–	–	–
		Stool	–	–	–	–
		Serum	–	–	–	–
		Sputum	–	–	–	–
		Urine	–	–	–	–
	10	Throat swab	+		–	
		Stool	–		–	
		Serum	–		–	
		Urine	–		–	
	11	Throat swab	–	–	–	–
		Stool	–	–	–	–
		Serum	–	–	–	–
		Sputum	–	–	–	–
		Urine	–	–	–	–
	12	Throat swab		–	–	–
		Stool		–	–	–
		Serum		–	–	–
		Urine		–	–	–
	13	Throat swab		–	–	–
		Stool		–	–	–
		Serum		–	–	–
		Urine		–	–	–
	14	Sputum			–	–
	15	Throat swab			–	–
		Stool			–	–
2 (onset 12/26/2003)	6	Throat swab	–	+		
	7	Throat swab	–	–		–
	8	Throat swab		+		+
	9	Throat swab		–		
	10	Throat swab	–	–		
3 (onset 12/30/2003)	9	Throat swab	–	–		–
		Stool	–	–		–
		Urine	–	–		–
	10	Throat swab	–			–
	11	Throat swab		–		
4 (onset 1/8/2004)	12	Stool	–	–	+	–
	15	Throat swab	–	–	–	–
	16	Stool	–		–	–

^a^SARS-CoV, severe acute respiratory syndrome–associated coronavirus; RT-PCR, reverse transcription–polymerase chain reaction; +, positive, –, negative. Where no value appears, the test was not performed. ^b^Number of days the specimen was collected after symptom onset. ^c^Specimens tested by Government Virus Unit, Hong Kong Special Administrative Region, with a subset tested by Department of Microbiology, Queen Mary Hospital.

## Discussion

The participation of multiple laboratories in this study documented SARS-CoV infection in these patients and permitted comparisons of results obtained with different assays. Antibody testing provided a relatively early indication of SARS-CoV infection in all four patients, as early as 6 days but no later than 9 days after onset of illness. SARS-CoV antibodies have been reportedly detected as early as 6 days after onset, but they are more commonly detected after 10 to 14 days (*2**,**4**,**18*). Although this early appearance of antibody is consistent with antibody response seen during the 2003 SARS outbreak, it could also indicate differences from that outbreak. For example, these patients may have had a longer incubation period or may have been previously infected with a SARS-like coronavirus that primed their immune systems for a rapid anamnestic antibody response. A longer incubation period, possibly because of low virus inoculum or infection with a virus that replicated less efficiently, could have provided additional time to mount an antibody response, leading to an apparent rapid antibody induction.

SARS-CoV was not isolated from any patient, and viral RNA was detected in only 3 of 4 patients at relatively low levels. This finding is consistent with results from studies during the 2003 outbreak that suggested high virus titers are associated with more severe illness and more efficient virus transmission (*19**,**20*). All of the patients survived, and none showed evidence of transmission to others. Only one stool specimen from one of these patients (patient 4), collected during week 2 of illness, was positive for SARS-CoV RNA. This finding contrasts with reports from the 2003 outbreak that a high percentage of stool specimens collected during week 2 of illness were positive for SARS-CoV by RT-PCR and that stool specimens were more likely to be positive than other specimens during week 2 of illness (*4*). The low virus titer found in these patients may reflect infection acquired directly from animals, before the virus acquired genetic changes that facilitate infection in humans. Sequence data recently reported by the Chinese SARS Molecular Epidemiology Consortium (*21*) suggest that SARS-CoV may have adapted to humans during the 2003 outbreak. Authors noted that the S protein gene had a higher ratio of coding to noncoding changes in the early stages of the outbreak, compared with later stages. This finding suggests a selective advantage for these coding changes (presumably related to infection in humans) and is consistent with findings from other coronaviruses that amino acid changes in the S protein can affect tissue tropism and disease associated with infection (*22*).

A previously unrecognized concern is the potential for serologic cross-reactions between human coronaviruses OC43 and 229E and the SARS-CoV. During the course of the workup of these patients, one laboratory showed an antibody response to both SARS-CoV and OC43 in one patient. This reaction was unexpected and required further testing to definitively determine which virus induced the antibody response. Subsequent neutralization and EIA antibody results demonstrate that SARS-CoV, not OC43- or 229E-like coronaviruses, induced the antibodies detected. The weight of the evidence therefore suggests that these patients were infected with SARS-CoV and not OC43- or 229E-like coronaviruses. This finding reinforces the need to better understand mechanisms underlying apparent cross-reactions between SARS-CoV and other human coronaviruses with nonneutralization serologic assays.

These cases illustrate the diagnostic difficulties that can occur in evaluating patients for SARS. The ability to confidently confirm or negate a diagnosis allows control efforts to focus on the most important cases and minimizes unnecessary social and economic disruption. The cost of missing a case can be high if further spread occurs, and the cost of false-positive diagnosis to the patient, family members, healthcare facility, and community can also be substantial.

In response, WHO established the SARS Reference and Verification Laboratory Network, which verifies all suspected cases of SARS-CoV infection outside the country in which the cases occur (*11*). WHO and other groups have also begun to provide test samples and proficiency panels that allow laboratories to assess their assays' performance and guidelines for specimen management to minimize the chance of contamination. The rapid identification and confirmation of SARS-CoV infection in these four cases exemplify the successful collaboration between local and WHO Network Laboratories and highlight the importance of continued cooperation in the event of the appearance of new SARS cases.
